# Bifunctional of Fe_3_O_4_@chitosan nanocomposite as a clarifying agent and cationic flocculant on different sugar solutions as a comprehensive semi industrial application

**DOI:** 10.1038/s41598-024-52111-6

**Published:** 2024-01-22

**Authors:** Hemat M. Dardeer, Ahmed S. Ibrahim, Ahmed N. Gad, Abdel-Aal M. Gaber

**Affiliations:** 1 Chemistry Department, Faculty of Science, South Valley University, Qena, Egypt; 2 Faculty of Sugar and Integrated Industries Technology, Assiut University, Assiut, Egypt; 3 Research and Development Center of ESIIC, Quos, Egypt; 4 Chemistry Department, Faculty of Science, Assiut University, Assiut, 71516 Egypt

**Keywords:** Environmental sciences, Materials science

## Abstract

In the sugar industry, eliminating side impurities throughout the manufacturing process is the most significant obstacle to clarifying sugar solutions. Herein, magnetic chitosan (MCS) nanocomposite was Fabricated to be used as a biodegradable, environmentally friendly clarifying agent throughout the cane juice and sugar refining processes. Fe_3_O_4_ was synthesized using the coprecipitation procedure, and then MCS was combined using a cross-linking agent. Furthermore, 14.76 emu g^−1^ was the maximum saturation magnetization (Ms) value. Because MCS is magnetically saturated, it may be possible to employ an external magnetic field to separate the contaminant deposited on its surface. Additionally, zeta potential analysis showed outstanding findings for MCS with a maximum value of (+) 20.7 mV, with improvement in color removal % up to 44.8% using MCS with more than 24% in color removal % compared to the traditional clarification process. Moreover, utilizing MCS reduced turbidity from 167 to 1 IU. Overall, we determined that MCS nanocomposite exhibits considerable effectiveness in the clarifying process for different sugar solutions, performing as an eco-friendly bio-sorbent and flocculating material.

## Introduction

A crucial industrial cash crop, sugarcane serves as the main source of raw materials to produce sucrose, alcohol, and bioenergy. Usually, mechanical pressing is used to remove the sugarcane juice from the cane stems. Natural sugarcane juice is full of vitamins, minerals, amino acids, flavonoids, and sugars (sucrose, glucose, and fructose), as well as phenolic acids (ferulic acids, coumaric acids, caffeic acids, ellagic acids, and gallic acids), and amino acids^1–3^. In the most heterogeneous sugarcane juice, there are multiple non-sugar colloids made up of protein, starch, pectin, gum, wax, and particles of clay^4^.

White sugar quality has long been determined by the color of sugarcane juice^5^. The current traditional methods for the decolorization of white sugar include sulfitation and carbonation. The residual sulfur component of the sugar produced is relatively large, notwithstanding the convenience of use, low production costs, and high occupancy of the sulfurous acid process^6^. Contrarily, the quality of white sugar made using the carbonic acid process is superior to that made using the sulfite method, but there are disadvantages as well, including high production costs and challenging alkaline waste treatment^7^.

Since clarification has a major effect on both sugar yield and quality, therefore, it is important to improve it to enhance the quality of sugar. To generate clear juice (CJ) with the lowest concentration of soluble and insoluble contaminants to maximize sugar production, clarifying sugar cane juice is fundamentally intended to do this^8^. Refining is the removal of impurities and color compounds from raw sugar to produce a product that is as close to being 100% pure sucrose as possible^9^. This process is primarily made up of a set of unit operations, with the clarification and color removal stages, and depends on the quality and characteristics of the raw sugar^10^.

Polyacrylamide is the most widely used flocculant in the sugar industry to improve separation during the clarification process, refineries that use the phosphatation process to improve the separation of calcium precipitates from the sugar solution, and the production of raw sugar. Studies and research on the topic of sugar solution flocculation would serve as the foundation for studies on the usage of polyelectrolytes to improve color removal in the sugar industry processes^11,12^.

Due to its low cost, availability, abundance, biodegradability, biocompatibility, nontoxicity, and high adsorption capacity for its ability to remove organic pollutants from wastewater, chitosan (CS) and its derivatives have attracted a lot of interest. However, they have substantial drawbacks that limit their effectiveness in the adsorption process, such as poor mechanical characteristics, weak acid stability, low surface area, low porosity, and low temperature resistance^10^.

Chitosan can be combined with other substances to create composite adsorbents, which is an excellent way to get around some of the disadvantages of chitosan and enhance its adsorption capacity^10^. In general, ferromagnetic materials, alloys, oxides, or composite structures based on iron, cobalt, and nickel are referred to as magnetic particles. The most prevalent and widely used type of naturally occurring iron oxide is magnetite, which has the chemical formula Fe_3_O_4_^13^. The ferrous ions occupy half of the octahedral lattice sites, ferric ions occupy the other half, and all of the tetrahedral lattice sites, defining its crystalline cubic inverse spinel structure^14^. A chitosan polymer matrix and a dispersed phase containing magnetic particles make up a magnetic chitosan composite (MCS).

As a result of its important biological and chemical characteristics, chitosan has undergone extensive research as a foundation material for magnetic carriers. Recent years have seen a surge in interest in MCS due to its better performance, which has led to substantial study into the production and use of these materials in several scientific domains^12,15^. MCS materials have a wide range of biomedical, environmental, and analytical applications in the biomedical, environmental, and analytical fields, including applications for enzyme-based biofuel cells, anti-cancer embolotherapy, targeting drug carriers, artificial muscle, bone regeneration, and fluorescence probes^16^. Additionally, MCS is frequently employed in the processes of clarifying sugar and treating wastewater^17^. MCS has a high adsorption capacity and a quick adsorption rate compared to other adsorbents, even at low concentrations and rapid equilibrium durations. Chitosan-based adsorbents can be challenging to remove from the aqueous solution after adsorption using conventional separation techniques like filtration and sedimentation because adsorbents may clog filters or be lost.

Recent research has concentrated on magnetic separation technology to address issues with the simplicity of separation and regeneration of adsorbents. An alternate technique for treating water and wastewater that has recently drawn a lot of attention is separation technology using magnetic adsorbents^18,19^. In the 1970s and 1980s, scientists started to realize that magnetic materials may be used to separate metal contaminants from various matrices that are magnetic field sensitive. MCS are primarily made of an inorganic metal oxide and chitosan organic material. Chitosan generally lacks magnetic characteristics, so for the particles to perform best in particle separation, a separate magnetic component must be introduced^19^.

## Materials and methods

### Materials

Chitosan purchased from Alpha Chemika, India with DA degree = 82.4% and high molecular weight. Ferrous sulfate heptahydrate (FeSO_4_·7H_2_O, 99%) was purchased from Piochem, Egypt, as green crystals. Ferric chloride hexahydrate (FeCl_3_·6H_2_O, 97%) was purchased from Piochem, Egypt. Epichlorohydrin was purchased from Loba Chemie, India. Glacial acetic acid (CH_3_COOH) purchased from Fischer Scientific, 99.5% pure. Phosphoric acid (H_3_PO_4_, 70%) was purchased from El-Nasr Co. for intermediate chemicals. Anionic flocculant, Magna floc-LT27 purchased from Solenis Chemicals India. Calcium oxide (CaO, 85%) was brought from El-Minia quarries. Mixed Juice (MJ) was brought in direct line from Quos sugar factory after milling. Raw sugar was brought from the factory as unconfirmed white sugar to obtain sugar syrup.

### Preparation of MCS nanocomposite

**Figure 1:**
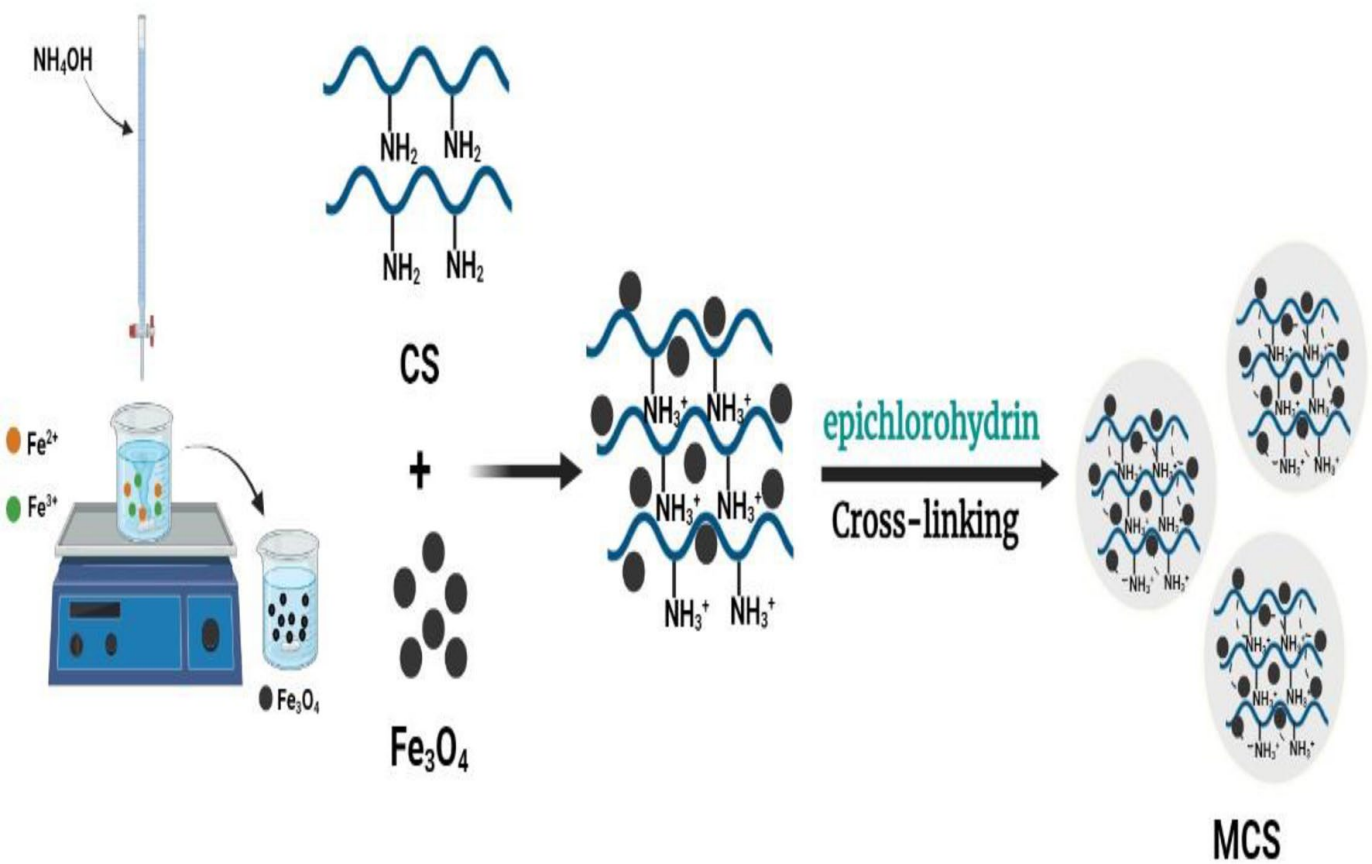
Preparation mechanism of MCS nanocomposite.

Magnetic chitosan nanocomposite was fabricated using the coprecipitation method and cross-linking agent. Firstly, 50 mL of distilled water was added to 4 g of FeCl_3_·6H_2_O and 3 g of FeSO_4_·7H_2_O, then the mixture was stirred for 10 min while raising the temperature to 70 °C, after that 2 M of ammonium hydroxide solution was slowly dripped into the mixture until forming magnetite (Fe_3_O_4_) as shown in Fig. 1. Subsequently, 10 g of chitosan was added to 1 L of 1% (v/v) acetic acid solution and stirred on a magnetic stirrer for 22 h at 800 rpm until chitosan was completely dissolved. After that, the formed magnetite was added to the chitosan solution under stirring for another 12 h to obtain a uniform homogenous suspension. Then 5 mL of epichlorohydrin was added dropwise to avoid the formation of big bulk colloidal precipitation until the crosslink was performed for MCS as shown in Fig. 1.

### Semi-industrial application

Studying the impact of MCS nanocomposite on flocculation and color removal requires utilizing the phosoflotation (phosphatation followed by flotation) process in the case of sugar syrup, and the traditional phosphatation process in the case of sugar juice. Our experiments were conducted at Quos Pilot Plant in Egypt and were designed to study the effect of MCS nanocomposite on both flocculation and color removal, as well as identify the best conditions for the clarification process. The semi-industrial experiment design was scientifically proposed by Eggleston at Audubon Sugar Institute^20^. Firstly, for sugarcane juice, 60 L of mixed juice (MJ) were first transported from the plant and divided into three reaction tanks (20 L/tank), which were then heated to 75 °C to begin the clarifying process as follows: (i) traditional phosphatation (control sample) and (ii) traditional phosphatation with MCS nanocomposite. Samples were separately added to reaction tanks with identical conditions, 5% H_3_PO_4_ (300 ppm P_2_O_5_), and Ca(OH)_2_ baume (6) until the pH was 7.3 ± 0.1. The temperature is then increased until flashing at 103 °C before the anionic flocculant is added (dosage: 4 ppm). After the flocs had finally settled in the clarifier (retention time: 1 h), samples of clear juice (CJ) were taken for the testing of quality parameters.

Secondly, for sugar syrup, under the same conditions, 60 L of sugar syrup was made at 62 ± 1 °Brix and divided into three reaction tanks (20 L each tank). Once the temperature reached 80 °C, 480 mL of 5% H_3_PO_4_ (~ 350 ppm P_2_O_5_) and 400 mL of Ca(OH)_2_ baume (6) were added, respectively, until the pH reached 7.1 ± 0.1, which corresponded to the control sample. After that, the synthesized nanocomposite was added to the respective tank (dose = 200 ppm), followed by the flotation process using the aeration pump for 30 s, and the addition of 140 mL of an anionic flocculant (dose 10 ppm). The flocs were then allowed to float for approximately 25 min, after which the samples of clear syrup were collected for investigation of quality parameters.

### Analysis and instrumentations

The crystalline structure of each material and its composite were determined by powder X-ray diffraction (XRD) which was recorded using Philips diffractometer (Model PW 2103, λ = 1.5418 Å, 35 kV and 20 mA) to obtain the crystal structures of the material in the 2θ region at 10° to 70°. Fourier Transform Infrared spectra (FT-IR) were measured using a Shimadzu-470 spectrophotometer, in the wavenumber range of 400–4000 cm^−1^ using KBr tablet to assign different functional groups contained in the synthesized samples. The morphological properties and compositions of CS and MCS nanocomposite were studied using Scanning Electron Microscope (SEM) and imaging was carried out using JSM T200 (JEOL, Japan) to observe the surface configuration and morphologies. Transmission Electron Microscope (TEM) and imaging was carried out using JEM 100 CXII (JEOL, Japan). Vibratory sample magnetometer (VSM, Lakeshore 7410) was carried out at room temperature using a vibrating sample magnetometer equipped with 2T magnet and under an applied magnetic field of 20 kOe to obtain the magnetic properties of the samples. Thermogravimetric analysis (TGA) was carried out using (Shimadzu DTG-60H, Japan) to investigate the thermal stability of MCS composite. Zeta potentials and particle size distribution of CS and MCS were determined by using a zeta potential and nanoparticle analyzer (Nano-ZS90X, Mal*v*ern, England). The compositions on the surface of MCS were analyzed by using an X-ray photoelectron spectroscopy (XPS), K-Alpha (Thermo Fisher Scientific, USA) with monochromatic X-ray AI K-alpha radiation -10 to 1350 e.v spot size 400 μm. Color absorbance was obtained using Jenway 7310 spectrophotometer.

## Results and discussion

### Characterization of MCS nanocomposite

#### SEM and TEM analyses

**Figure 2:**
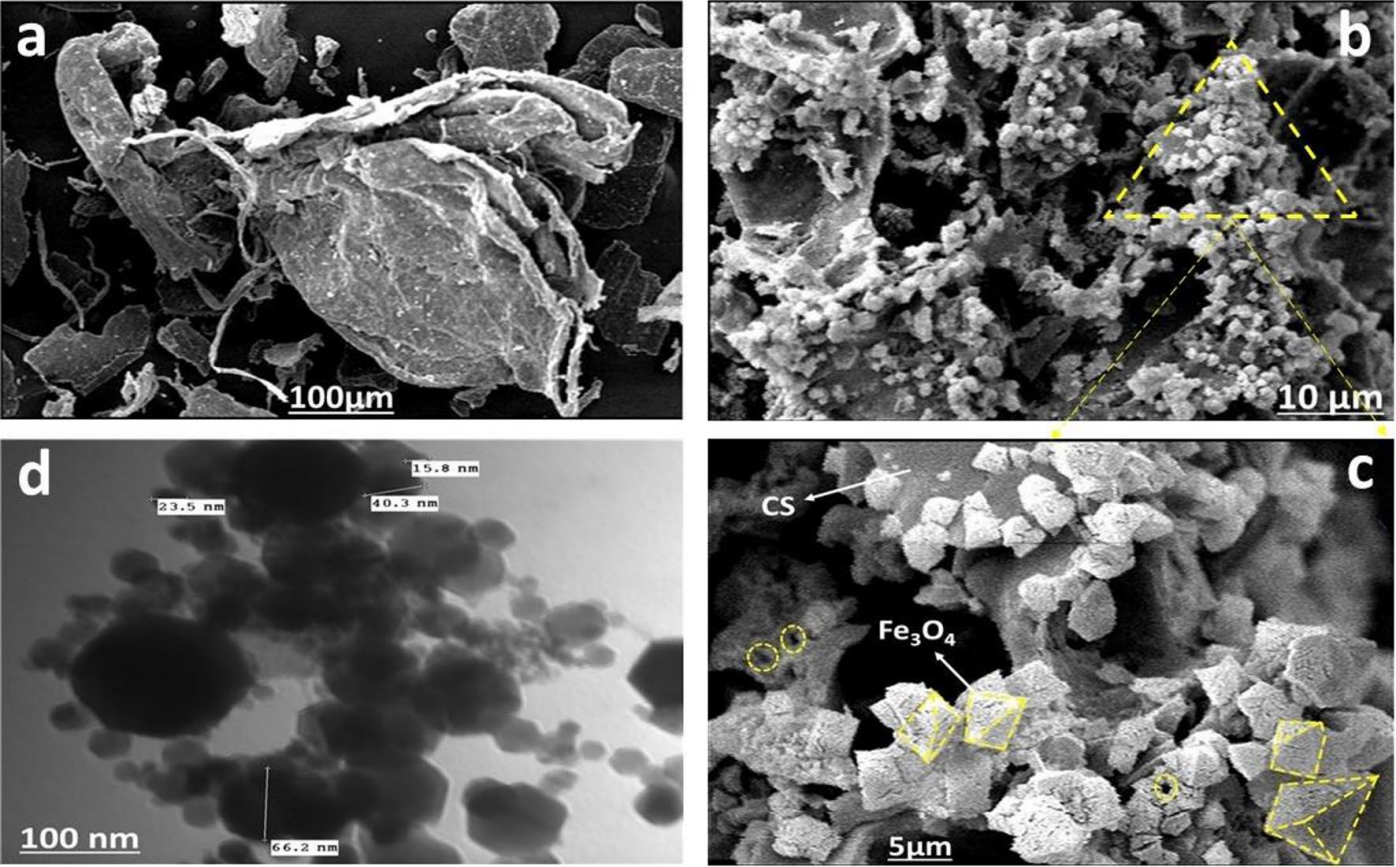
(**a**) SEM image for CS, (**b**) SEM image for MCS, and (**c**) TEM image for MCS.

Every image was found to have a unique texture and morphology, as seen in Fig. 2a, which depicts the morphology of CS, which has certain protrusions on its surface^21^. As can be seen in Fig. 2b, SEM image of MCS reveals that the sample has nearly uniform grains of magnetite crystals on the surface of the chitosan, which display regular octahedrons with rough surfaces. The low-magnification SEM image suggests that it may be the result of magnetic iron oxide particles being embedded within the cross-linked chitosan. Further evidence linking CS to microspheres relatively smooth surface suggests that the Fe_3_O_4_ particles are tightly wrapped in chitosan polymers, preventing the magnetic carriers from dropping out^12^. A great number of slit pores can be seen on the MCS surface in the high-magnification SEM image as shown in Fig. 2c. In addition to being a material with a high surface area, its porous structure may provide more adsorption sites and enhancing MCS's capacity to adsorb anionic dyes in sugar solutions^22^. Nearly spherical and hexagonal nanoparticles with an average diameter of about 40 nm are visible in the sample’s TEM image. Further proof that a core–shell and chain-like structure with good dispersion have successfully produced is provided by MCS nanocomposite^23^, as illustrated in Fig. 2d. Additionally, in conformity with SEM images, CS has been successfully coated onto the surface of the Fe_3_O_4_ crystals. Also, a TEM picture revealed that the surface of MCS is made up of organized channels with hexagonal pore structures^24^. On the other hand, because of the nanometer-scale particle size, the adsorbents may have a large specific surface area. Similarly, the good dispersion of MCS nanocomposite can increase the surface area in contact with colorants substrate and contaminants, which will increase its adsorption capacity^25^.

#### FTIR analysis

**Figure 3:**
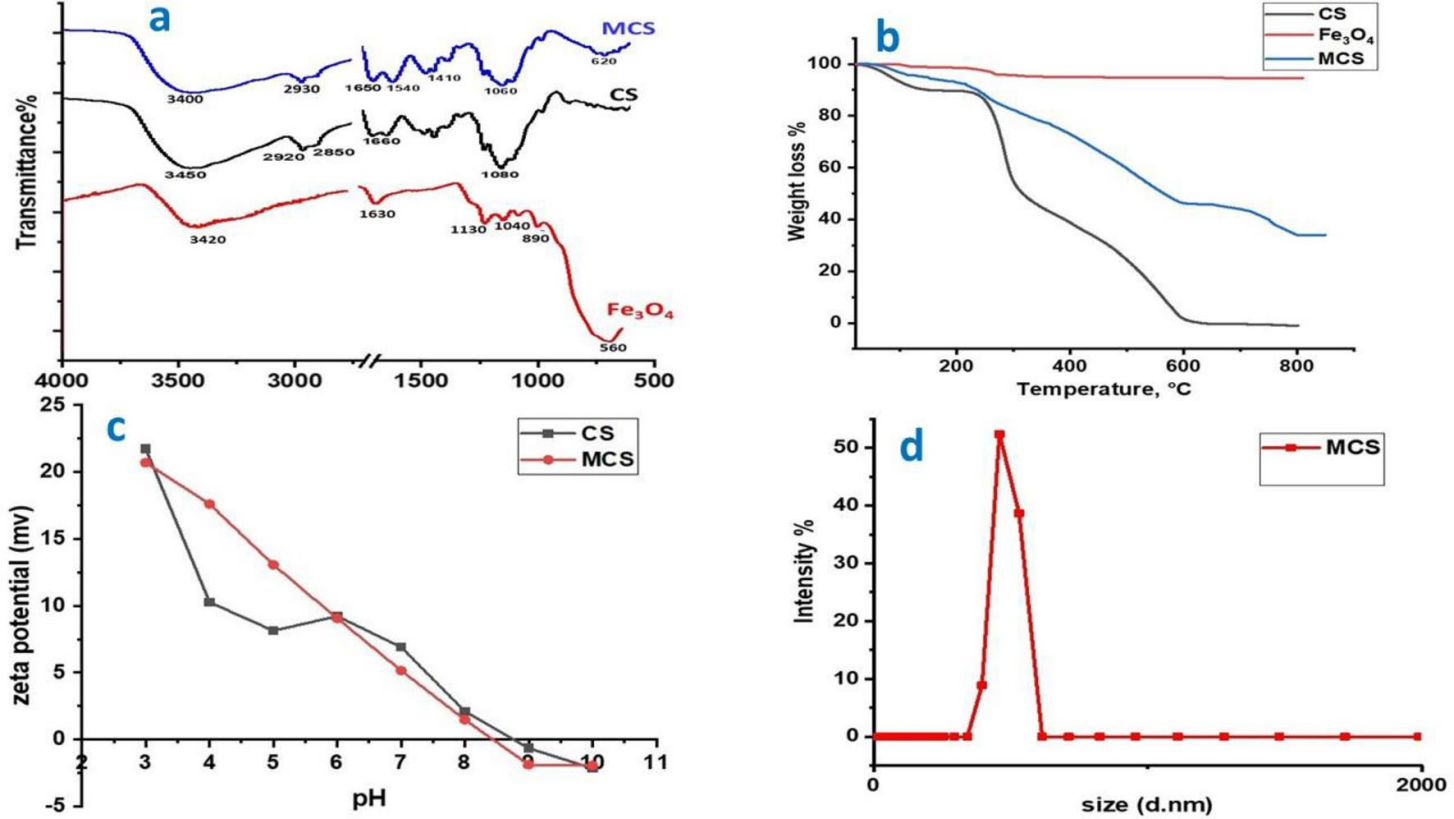
(**a**) FT-IR spectra of CS, Fe_3_O_4_, and MCS nanocomposite, (**b**) Thermogravimetric curves for CS, Fe_3_O_4_, and MCS nanocomposite, (**c**) Zeta potential curves for CS and MCS nanocomposite, and (**d**) Particle size distribution of MCS nanocomposite.

As shown in Fig. 3a, pure Fe_3_O_4_ nanoparticles, produce a distinctive peak at 560 cm^−1^, which was attributed to the Fe–O stretching mode in the tetrahedral state of Fe_3_O_4_^12^. The FTIR spectrum of MCS also exhibits the reflection of the typical peaks related to chitosan and Fe_3_O_4_ nanoparticles, albeit with a small peak shift. In particular, there has been a shift in the peak at 620 cm^−1^ from 560 cm^−1^, which corresponds to the stretching vibration of Fe–O. A further indication of the substantial interactions (such as metal coordination and hydrogen bonding) between the Fe_3_O_4_ nanoparticles and chitosan during MCS formation is the shift of the peak corresponding to the stretching vibration of N–H^26^ from 1660 to 1650 cm^−1^. The MCS composite strengthened the typical peaks of –OH and –NH_2_ at 2930 and 3400 cm^−1^, respectively. The abundant polar groups in chitosan, notably –NH_2_ and –OH, interacted polarly with the Fe–O bond and contributed to this outcome by encouraging the coupling of polar groups. The results of the FTIR spectra revealed that magnetic nanoparticles were coated with this polymer as a result of the precipitation interactions with chitosan^26^.

#### TGA analysis

Since the TGA is employed in order to determine the weight percentage of different components in composite particles and to assess the thermal stability of the composite. The TGA curves of Fe_3_O_4_, CS, and MCS are shown in Fig. 3b. The TGA thermogram of the MCS nanocomposite shows three separate weight losses and a thermal degradation profile that is comparable to that of chitosan. At temperatures between 100 and 150 °C, bound water vaporized, causing the initial stage of weight loss in MCS. The second stage, which exhibited significant weight loss, started at 200 °C and increased to 580 °C. The primary chain of chitosan molecules may have broken down as a result^27^. After 580 °C, the MCS's weightlessness started to balance progressively. Nevertheless, throughout the whole heating process (30–800 °C), Fe_3_O_4_ showed excellent thermal stability and lost nearly little weight. Chelation between Fe^3+^ and CS caused the reaction that linked Fe_3_O_4_ and CS together^28^. Additional MCS bridging and structural modifications to CS increased CS thermal stability in MCS nanocomposite. The temperature at which MCS finally decomposed was higher than the temperature at which pure CS decomposed. As a result, the Fe_3_O_4_ nanoparticles have been successfully functionalized with chitosan groups, according to TGA thermogram data.

#### Zeta potential and particle size analyses

Figure 3c displayed the estimation of the surface charge of MCS and zeta potential measurements as a function of pH indicating a noteworthy value for the positive charge of MCS nanocomposite. The protonation of amine groups on the particle surface is thought to be the cause of the positive zeta potential that exists below the isoelectric point (pI). The zeta potential of MCS reduced when the pH level increased as shown in Fig. 3c. Amine groups were protonated and obtained a positive charge in the form of -NH_3_^+^ at pH < 8.44, which is the isoelectric point (pI) for MCS. Thus at pH < 8.44, MCS demonstrated a positive potential. However, in the basic medium, a negative potential was seen at pH > 8.44. As a result, MCS was a modified adsorbent with the ability to adsorb cations in alkaline conditions and anions in an acidic environment^12^. The maximum value for the MCS nanocomposite was (+) 20.7 mV because the magnetite negative charge neutralized the charge of the quaternary ammonium group. Besides the material's ability to function as an adsorbent, the performance of the material in decolorization with anionic colorants is still obvious and promotes these interactions with quaternary ammonium groups of the nanocomposite. Additionally, the prepared MCS nanocomposite was assessed for its particle size as shown in Fig. 3d. According to results from transmission electron microscopy, the particle size distribution displayed a unimodal curve, with the majority sizes ranging from 350 to 620 nm and a cumulative distribution peak at 415 nm and a diameter of 44 nm^29^.

#### XRD analysis

**Figure 4:**
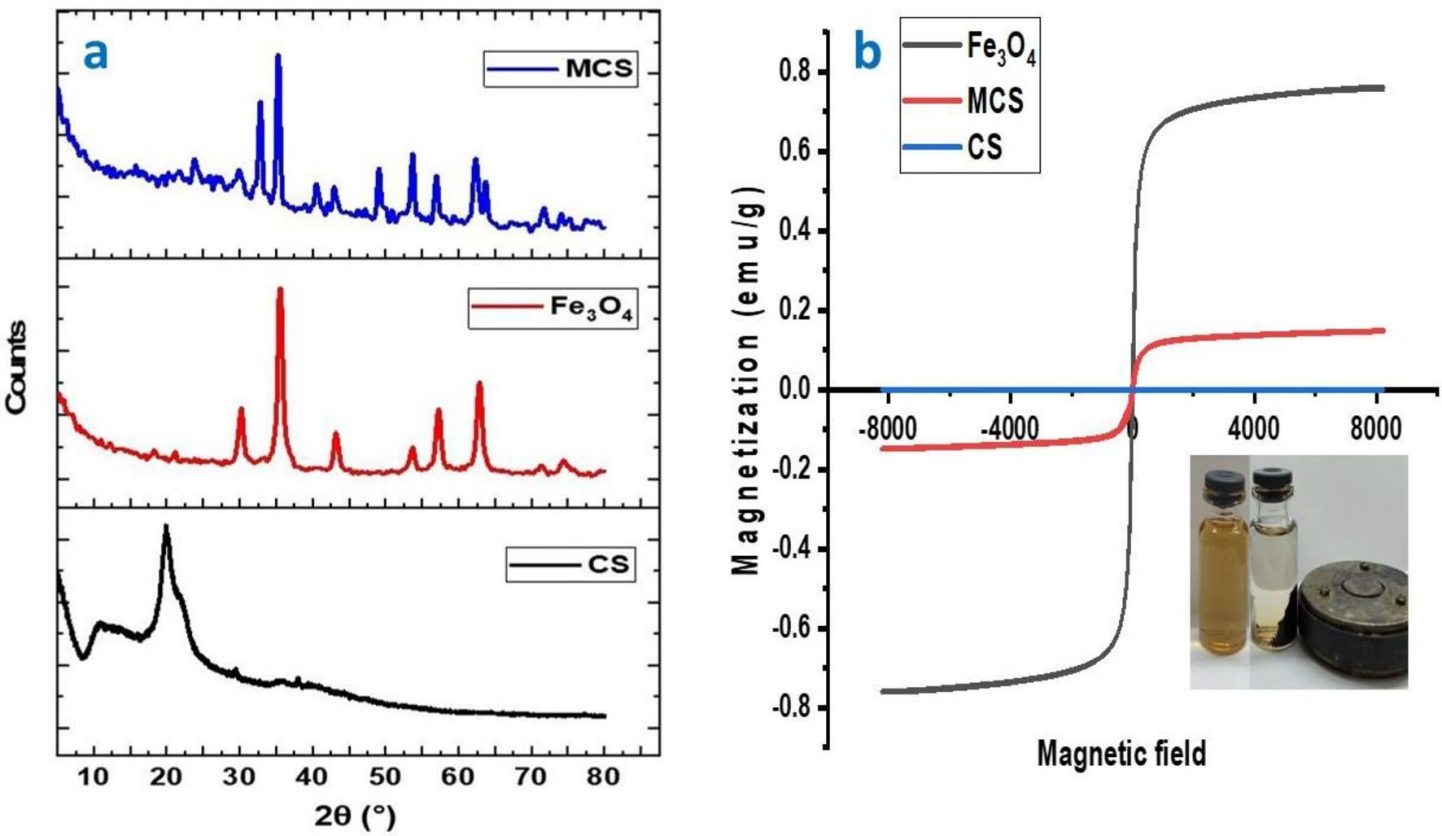
(**a**) XRD patterns of CS, Fe_3_O_4_, and MCS nanocomposite, and (**b**) Magnetization curves for Fe_3_O_4_ and MCS nanocomposite.

Chitosan had typical semi-crystalline characteristics (110) at its diffraction peak at 23.1°, as illustrated in Fig. 4a. The cubic spinel structures of the pure Fe_3_O_4_ particles had strong diffraction peaks at 30.28°, 35.62°, 43.3°, 53.8°, 57.4°, and 62.9°, which were consistent with the magnetite database^30^ and corresponded to the various Fe_3_O_4_ lattice planes (220), (311), (400), (422), (511), and (440), respectively. The relative intensity changed due to the change in the 2 signals in the XRD image of MCS. As an illustration, the peak at 20.13° was incredibly feeble, which may have been attributed to the quantity of chitosan supplied and the coprecipitation process of MCS^12^. The peaks splitting in MCS patterns at 40° and 80° are an indication of phase transformation that may be caused because of the dislocation in the crystal lattice of magnetite which confirms the formation of MCS nanocomposite. Additionally, both CS and Fe_3_O_4_ crystalline characteristic peak intensity dropped, these findings indicated the formation of MCS.

### Magnetization properties for MCS nanocomposite

Fe_3_O_4_ can be added to the material to give it magnetic characteristics. For magnetic material recovery and reuse, magnetic characteristics are necessary, to evaluate the magnetic separation capability of MCS, magnetic strength could be employed^12^. As seen in Fig. 4b, the magnetic hysteresis loop of the MCS exhibited an S-like shape and overlapped. At room temperature, MCS had a maximum saturation magnetization (Ms) value of 14.76 emu g^−1^. This finding was less than the value of 76.124 emu g^−1^ of pure Fe_3_O_4_ due to the existence of non-magnetic CS layers. The produced MCS sample may be recovered and separated from reaction media using an external magnetic field since it is magnetically saturated^31^.

### XPS analysis

**Figure 5:**
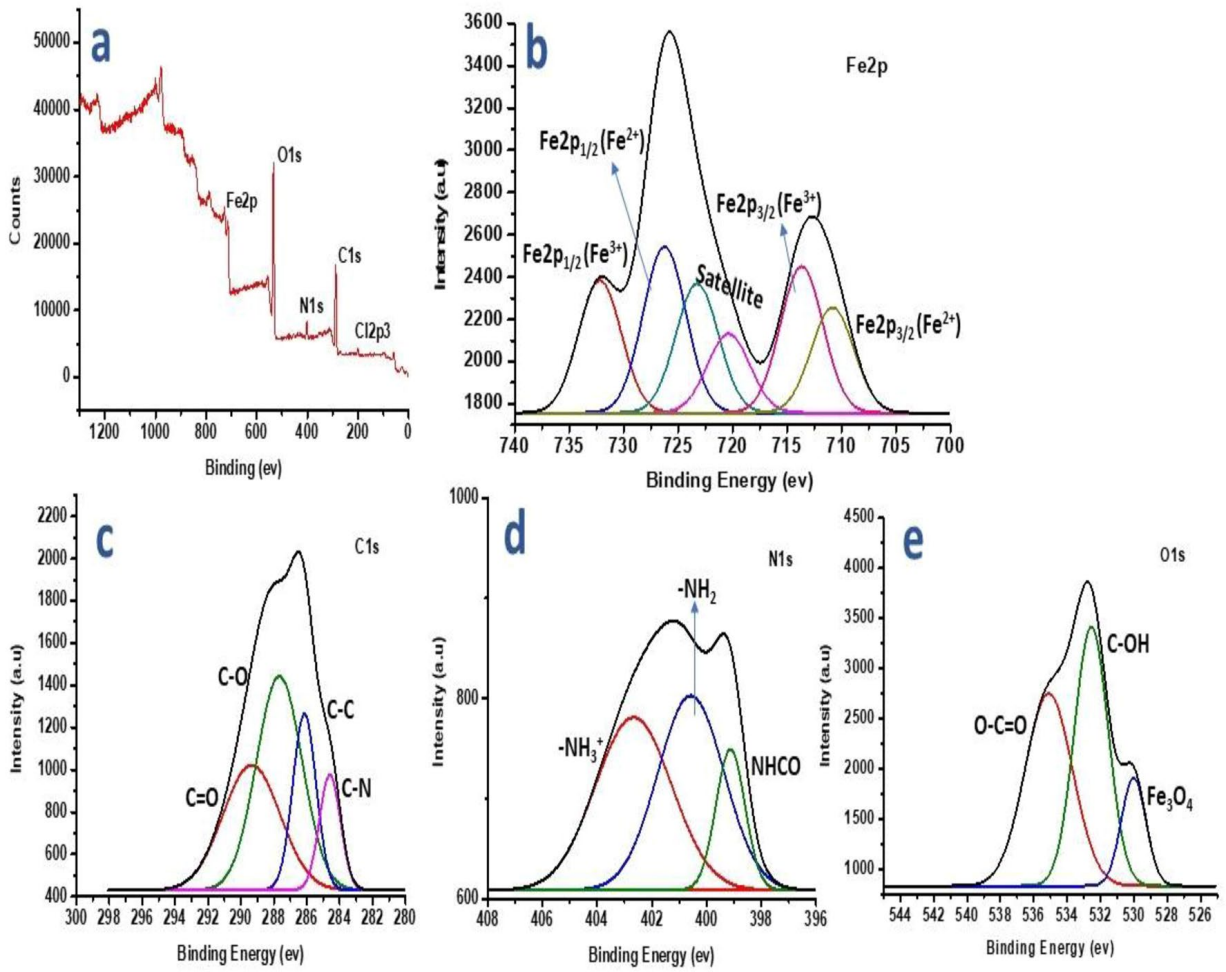
(**a**) XPS survey scan for MCS nanocomposite, (**b**) Fe2p spectrum of MCS nanocomposite, (**c**) C1s spectrum of MCS nanocomposite, (**d**) N1s spectrum of MCS nanocomposite, and (**e**) O1s spectrum of MCS nanocomposite.

A substance's surface chemical state and element composition can be thoroughly studied with XPS. The five primary elements Cl, C, N, O, and Fe were represented by the XPS survey scan for MCS nanocomposite in Fig. 5a, with signals of 200.64 eV, 288.23 eV, 402.26 eV, 534.13 eV, and 713.46 eV, respectively. The catalytic and magnetic properties of magnetite make it the most intriguing of the iron oxides. Surface reactivity and magnetism are denatured, and the crystal structure is changed from inverse spinel to spinel as a result of this reaction. Due to its sensitivity to Fe^2+^ and Fe^3+^ cations, X-ray photoelectron spectroscopy (XPS) measurement was used to establish the stability of the surface of the synthesized material to oxidation. The MCS fabricated composite contains Fe^2+^ (Fe 2p3/2: 710.65 eV, Fe 2p1/2: 725.79 eV and satellites: 717.76 and 722.39 eV), and Fe^3+^ (Fe 2p3/2: 712.72 eV, Fe 2p1/2: 729.2 eV), according to the high-resolution spectrum of Fe2p depicted in Fig. 5b^32^. The high resolution of C1s shown in Fig. 5c also displays four peaks at 284.88 eV, 286.29 eV, 287.74 eV, and 289.57 eV, which are associated with C–C, C–N, C–O, and C=O, respectively. Three fitting peaks with respective centers at 399.31 eV, 400.71 eV, and 402.17 eV were identified in the N1s spectrum presented in Fig. 5d as –NH_2_ (primary amine), –NH_3_^+^ (quaternary ammonium), and NHCO, respectively. Here, –NH_2_ has a molar proportion of 25.73%, and –NH_3_^+^ has a molar percentage of 7.97%, indicating a smaller positive value in the zeta potential analysis^27^. Furthermore, the Fe_3_O_4_ lattice oxygen atoms, the oxygen atoms of OH groups from surface vacancies, and O–C=O, respectively, are responsible for the peaks at 529.88 eV, 532.66 eV, and 535.37 eV in the high-resolution O1s spectra shown in Fig. 5e^10^.

### Results of application

Throughout the whole process of producing sugar, colorants constitute a major problem because they have a negative effect on crystallization and affect the quality of white sugar^8,10^. Phosphatation, sulfitation, and carbonation are the traditional clarification processes used in Egyptian factories to produce white and refined white sugar because the main objective of the sugar clarification process is the separation of impurities and coloring matters from raw sugar syrup. This study compared the color removal percentage and turbidity of clarified juice and syrup obtained from each clarification method, traditional phosphatation clarification (control), traditional phosphatation clarification with CS, and traditional phosphatation clarification with MCS on both sugarcane juice and the raw sugar syrup.

The comparisons were done on a semi-industrial scale. Thus, the use of novel green biodegradable nanocomposite in the semi-industrial sugar clarifying process for both sugarcane juice and refined sugar will be covered in this section.

### Evaluation of MCS nanocomposite on cane mixed juice (MJ)

The objective of this study is to improve the phosphatation clarification process efficiency by introducing a new green biodegradable clarifying agent. Accordingly, each case’s quality parameters such as brix, purity, turbidity, dissolved solids (DS), and color removal % percentage will be evaluated in order to determine the effectiveness and impact of the new clarifying agent on the clarification process^20^. A comparison of the color removal percentage and turbidity was conducted on clear juice (CJ) was obtained from each clarification method as currently phosphatation clarification (control), currently phosphatation clarification with CS, and currently phosphatation clarification with MCS on Egyptian MJ.

**Figure 6:**
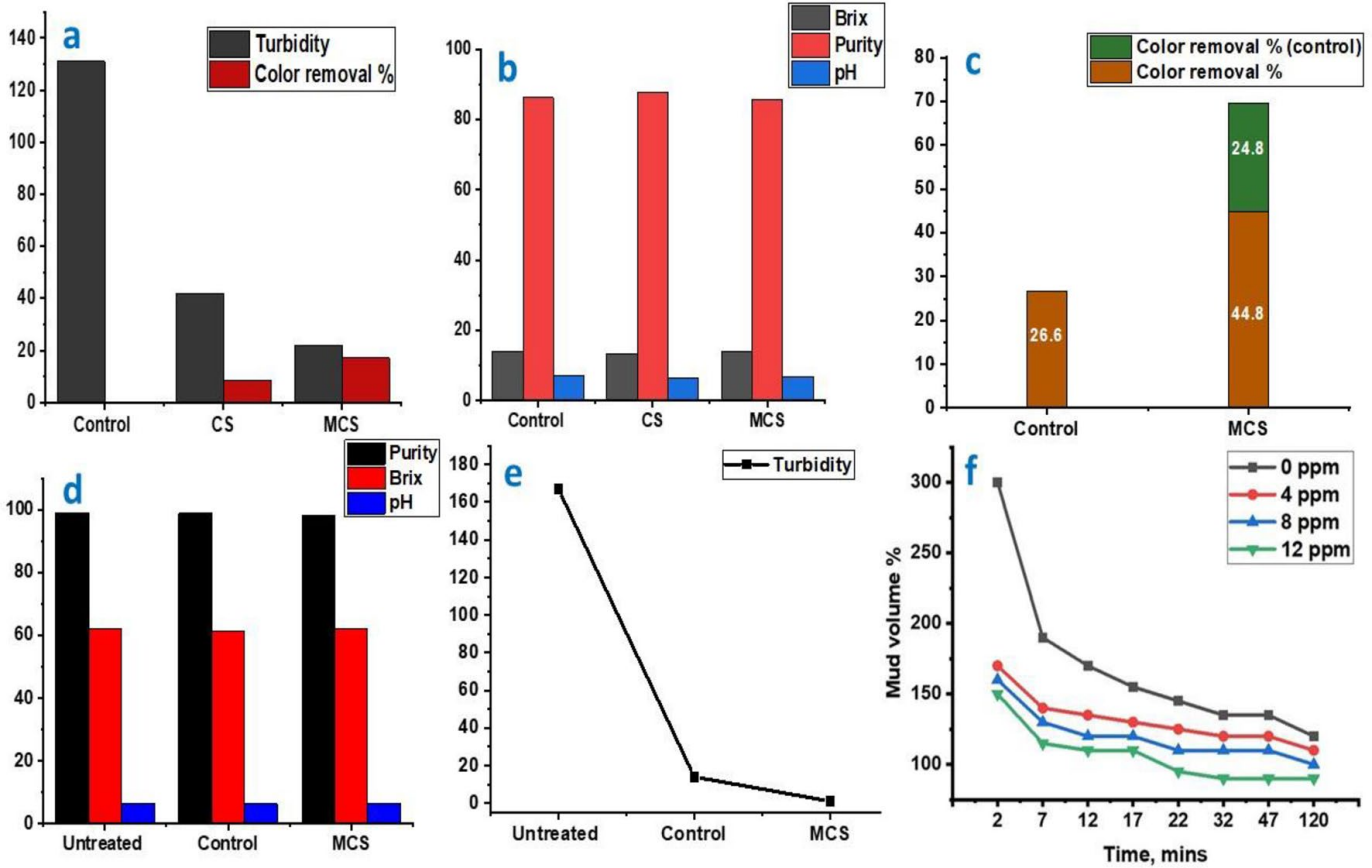
(**a**) Comparison between control, CS, and MCS in color removal % and turbidity on CJ, (**b**) Brix°, purity, and pH performance for control, CS, and MCS on CJ. (**c**) Comparison between control and MCS in color removal % on sugar syrup. (**d**) Brix°, purity, and pH performance for untreated syrup, control, and MCS. **(e)** Turbidity curve for untreated syrup, control, and MCS. (**f**) Flotation rate curves for anionic flocculant dosages against time.

For more accurate evaluation three replicates were carried out at a pilot scale in Quos sugar factory, and for each trial average was calculated Due to an increase in active positive sites (quaternary amine) in the case of CS and its composites, as demonstrated in Fig. 6a, the color removal % increased to 8.7 and 17.1 using CS and MCS, respectively compared to the control sample (currently phosphatation clarification). Furthermore, the morphological shape and cavity sites that are present in MCS nanocomposite as shown in Fig. 2c, enhance to capture impurities and color materials by forming an insoluble matrix that is mostly the result of electrostatic attraction and may be improved by the tiny slots that are formed when CS and Fe_3_O_4_ crosslink together^33^. As illustrated in Fig. 6b, it was demonstrated that applying MCS nanocomposite increased pH values compared to CS by itself since the protonated amine groups may interact electrostatically when the composites are formed via both inter and intra-molecule H-bonds^10^. The lower value of pH in CJ using CS and MCS nanocomposite is due to dissolving it in 1% acetic acid as presented in Table 1. While brix° and purity for CJ did not noticeably change while utilizing CS and MCS nanocomposite as shown in Fig. 6b.

**Table 1:** Comparison between control, CS, and MCS on the quality parameters of CJ.

Parameter	Untreated	Control	CS	MCS
Brix	13.80	13.96	13.44	14.02
Purity	84.18	86.19	87.75	85.75
Color% Brix	–	10,422	9516	8644
Turbidity	–	131	42	22
TDS	2.95	3.06	3.23	3.11
pH	6.09	6.98	6.59	6.85
Color removal %	–	–	8.7	17.1

### Evaluation of MCS nanocomposite on sugar syrup

**Figure 7:**
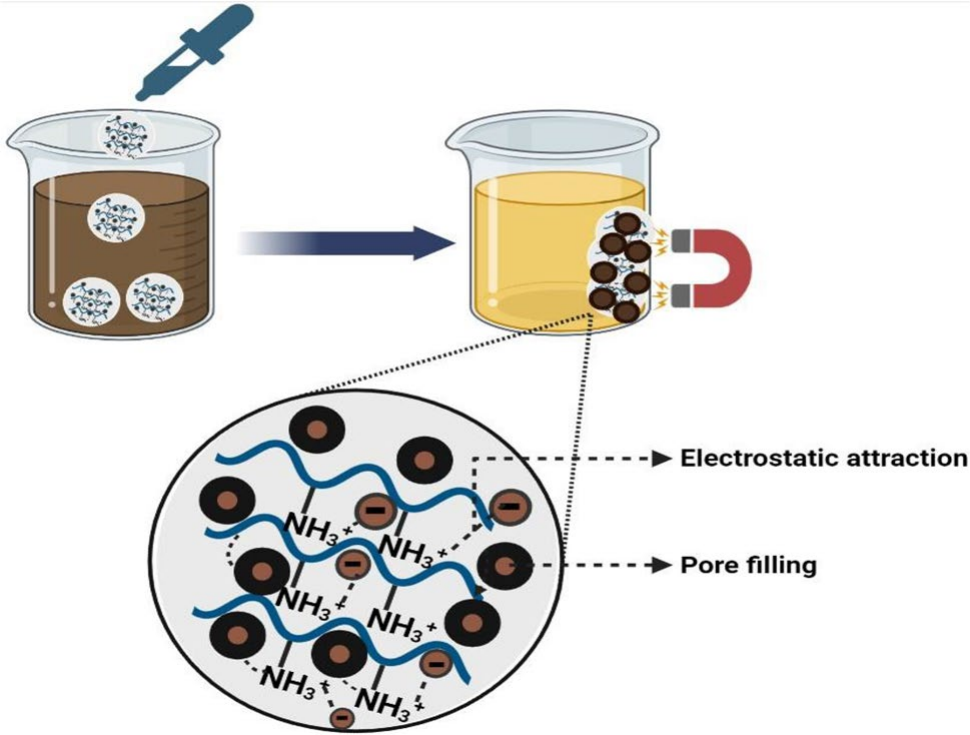
Adsorption illustration of colorants using MCS nanocomposite and magnetic field.

Here, the performance of the fabricated composite during the clarification process will also be evaluated by comparing the color removal percentage, turbidity, and flotation rate during the refining sugar process. Additionally, the clear syrup that was obtained was assessed using each of the following methods of clarification: currently phosphatation clarification (control) and currently phosphatation clarification with MCS on raw sugar syrup. Three replicates were conducted at pilot scale in Quos sugar factory, and each trial's average was determined. The color removal percentage utilizing MCS nanocomposite appears to have increased from 26.6 to 44.8% when compared to the control, as indicated in Fig. 6c. This increase can be attributed to the increased number of active sites (quaternary ammonium) as well as the channels and cavity structure previously discussed^34,35^. Furthermore, morphological shape results in the capture of impurities and the adsorption of coloring materials from the syrup in addition to a strong electrostatic interaction with the anionic colorants. Also, magnetic chitosan adsorbents are a great option for the decolorization process because of their facile magnetic separation and high chelating capacity^36^. Because of their larger specific surface area and porous shape, magnetic nanoparticles were used to construct the MCS nanocomposite adsorbent. In addition to the quaternarized ammonium groups that improve adsorption effectiveness, the protonation of FeOH to FeOH_2_^+^ may also contribute to an increase in electrostatic attraction. As a result, as illustrated in Fig. 7, MCS nanocomposite possesses both the magnetization and adsorption characteristics that enable the separation of the adsorbates and their recovery from reaction environments using an external magnetic field. Furthermore, Fig. 6c demonstrated a 24.8% improvement in color removal percentage when using MCS compared to the control, demonstrating the greater efficiency of using this green nanocomposite as shown in Fig. S8.

On the other hand, the pH values for untreated syrup, treated syrup using the traditional clarification process, and treated syrup with the synthesized clarifying agent are displayed in Fig. 6d, and no noticeable changes were found. This is a positive sign since the change in pH value can lead to sugar inversion and turn it into reducing sugars resulting in the formation of additional coloring material and increased sucrose losses. Figure 6e demonstrates a notable reduction in turbidity from 167 IU for the untreated syrup to 14 IU for the control and 1 IU with MCS as mentioned in Table 2. In addition to their role as cationic color precipitant, these studies demonstrated that MCS possessed cationic flocculation efficiency that improved the anionic flocculant to capture impurities present in sugar syrup^37,38^ as shown in Fig. S9. The performance of sugar syrup Brix° and purity before and after treatment is shown in Fig. 6d. The treated syrup using MCS showed a slight decrease in Brix° and purity values; this could be due to the strong electrostatic attraction between quaternary ammonium groups and coloring matters (soluble solids), as the zeta potential value for MCS nanocomposite is higher^39,40^.

**Table 2:** Comparison between traditional clarification and MCS on sugar syrup.

Parameter	Untreated	Control	MCS
Brix	62.28	61.50	62.17
Purity	99.1	98.9	98.4
Color% Brix	3324	2439	1834
pH	6.6	6.4	6.6
Color removal %	–	26.6	44.8
Color removal % (control)	–	–	24.8
Turbidity	167	14	1

### Effect of flotation rate

Studying the flotation rate of cationic flocculant is a key factor, as it determines the retention time for syrup to discharge. It's also critical for increasing white sugar productivity while lowering operating expenses. Thus, to reduce the dose of anionic flocculant without negatively affecting the flocculation process, the flotation duration has been investigated in this case to validate the effectiveness of the MCS nanocomposite as a cationic flocculant. The mud volume percentage for the flocs at various intervals with varying doses of anionic flocculant is displayed in Table 3. As can be witnessed in Fig. 6f, the mud volume% without anionic flocculant (0 ppm) is larger than the others, with its negative charge, the anionic flocculant quickly attracted the cationic flocs, forcing them to float to the surface of the syrup. As a result, the mud volume percentage increases, as seen in Fig. S9. Following the addition of anionic flocculant dosage for 120 min, as indicated in Fig. S10, the flocs stabilized after a certain time and the volume became stable without obviously changing. We can see from the results in Table 3 and Fig. 6f that even at a 4 ppm dosage of anionic flocculant, the flocculation process is still as effective as the dosage is 12 ppm.

**Table 3:** Mud volume percentages with anionic flocculant dosages.

Time (min)	Mud volume % with anionic flocculant dosages			
	0 ppm	4 ppm	8 ppm	12 ppm
2	300	170	160	150
7	190	140	130	115
12	170	135	120	110
17	155	130	120	110
22	145	125	110	95
32	135	120	110	90
47	135	120	110	90
120	120	110	100	90

## Conclusion

This study employed a new green clarifying agent as a cationic color precipitant and cationic flocculant in the clarification process. FTIR, XRD, and XPS investigations were used to confirm the formation of MCS nanocomposite. SEM and TEM images were used to highlight the morphological changes between the starting materials and the synthesized nanocomposite, ensuring that the green composite formed based on these changes in morphological forms. Additionally, the produced nanocomposite showed good thermal stability according to TGA study. Co-precipitation and cross-linking modification were used to synthesize MCS. Besides the chitosan amino group, the surface of MCS also included the crystal structure in the form of Fe_3_O_4_. As a type of paramagnetic material, MCS saturation magnetization was 14.76 emu g^−1^. These findings showed that Fe_3_O_4_ and chitosan had successfully modified MCS. The findings of the pilot-scale application demonstrated a notable increase in the percentage of color removal% when the synthesized nanocomposite was used instead of the traditional phosphatation method, which produced sulfur-less white sugar.

### Supplementary Information

Supplementary Information.

## Data Availability

The datasets used and/or analysed during the current study available from the corresponding author on reasonable request.

## References

[1] Meng L (2021). Understanding the pathways for irreversible aggregate clusters formation in concentrated sugarcane juice derived from the membrane clarification process. LWT.

[2] Kohli G (2019). Effect of non-thermal hurdles in shelf life enhancement of sugarcane juice. LWT.

[3] Abbas SR, Sabir SM, Ahmad SD, Boligon AA, Athayde ML (2014). Phenolic profile, antioxidant potential and DNA damage protecting activity of sugarcane (*Saccharum*
*officinarum*). Food Chem..

[4] Uchimiya M (2017). Roles of reversible and irreversible aggregation in sugar processing. Crit. Rev. Food Sci. Nutr..

[5] Zhang H, Luo J, Liu L, Chen X, Wan Y (2021). Green production of sugar by membrane technology: How far is it from industrialization?. Green Chem. Eng..

[6] Shi C (2019). Ceramic membrane filtration of factory sugarcane juice: Effect of pretreatment on permeate flux, juice quality and fouling. J. Food Eng..

[7] Cole M, Eggleston G, Wang YJ (2019). Understanding the causes of calcium carbonate crystal growth and inhibition during the carbonatation refining of raw sugars. Food Chem..

[8] Ibrahim AS, Gad AN, Dardeer HM, Gaber A-AM (2023). Chitosan-cellulose nanocomposite: Preparation, characterization, and evaluation as cationic color precipitant in sugar clarification process. Food Chem..

[9] Mostafa FA, Gad AN, Gaber AAM, Abdel-Wahab AMA (2022). Preparation, characterization and application of calcium oxide nanoparticles from waste carbonation mud in clarification of raw sugar melt. Sugar Tech..

[10] Ibrahim AS, Gad AN, Dardeer HM, Gaber A-AM (2022). Novel green biodegradable clarifying agents in sugar refining process using functionalized chitosan nanocomposites. Sustain. Mater. Technol..

[11] Fan S (2019). Magnetic chitosan-hydroxyapatite composite microspheres: Preparation, characterization, and application for the adsorption of phenolic substances. Bioresour. Technol..

[12] Song X, Chai Z, Zhu Y, Li C, Liang X (2019). Preparation and characterization of magnetic chitosan-modified diatomite for the removal of gallic acid and caffeic acid from sugar solution. Carbohydr. Polym..

[13] Lu Q, Choi K, do Nam J, Choi HJ (2021). Magnetic polymer composite particles: Design and magnetorheology. Polymers.

[14] Mohammadi H (2021). Synthesis and characterization of magnetite nanoparticles by co-precipitation method coated with biocompatible compounds and evaluation of in-vitro cytotoxicity. Toxicol. Rep..

[15] Hritcu D, Popa MI, Popa N, Badescu V, Balan V (2009). Preparation and characterization of magnetic chitosan nanospheres. Turk. J. Chem..

[16] Ma W, Ya FQ, Han M, Wang R (2007). Characteristics of equilibrium, kinetics studies for adsorption of fluoride on magnetic-chitosan particle. J. Hazard. Mater..

[17] Abou El-Reash YG, Otto M, Kenawy IM, Ouf AM (2011). Adsorption of Cr(VI) and As(V) ions by modified magnetic chitosan chelating resin. Int. J. Biol. Macromol..

[18] Linley S, Leshuk T, Gu FX (2013). Magnetically separable water treatment technologies and their role in future advanced water treatment: A patent review. Clean (Weinh).

[19] Reddy DHK, Lee SM (2013). Application of magnetic chitosan composites for the removal of toxic metal and dyes from aqueous solutions. Adv. Colloid Interface Sci..

[20] Eggleston G (2003). New Orleans, LA 701 24 2Cora Texas Manufacturing Co. Res. 32540 B Texas Rd. White Castle. LA 70788. J. Food Process. Preserv..

[21] Li D, Cui H, Hayat K, Zhang X, Ho C-T (2022). Superior environmental stability of gelatin/CMC complex coacervated microcapsules via chitosan electrostatic modification. Food Hydrocoll..

[22] Gouda M, Ibrahim HIM, Negm A (2022). Chitosan containing nano Zn-organic framework: Synthesis, characterization and biological activity. Polymers.

[23] Sharifi MJ, Nouralishahi A, Hallajisani A, Askari M (2021). Magnetic chitosan nanocomposites as adsorbents in industrial wastewater treatment: A brief review. Cellulose Chem. Technol.

[24] Mu W, Yu Q, Li X, Wei H, Jian Y (2017). Efficient removal of Cs+ and Sr2+ from aqueous solution using hierarchically structured hexagonal tungsten trioxide coated Fe_3_O_4_. Chem. Eng. J..

[25] Yismaw S (2019). Particle size control of monodispersed spherical nanoparticles with MCM-48-type mesostructure via novel rapid synthesis procedure. J. Nanopart. Res..

[26] Rathinam K, Kou X, Hobby R, Panglisch S (2021). Sustainable development of magnetic chitosan core–shell network for the removal of organic dyes from aqueous solutions. Materials.

[27] Guo LY (2021). Quaternary ammonium-functionalized magnetic chitosan microspheres as an effective green adsorbent to remove high-molecular-weight invert sugar alkaline degradation products (HISADPs). Chem. Eng. J..

[28] Ates B (2018). Magnetic-propelled Fe_3_O_4_-chitosan carriers enhance l-asparaginase catalytic activity: A promising strategy for enzyme immobilization. RSC Adv..

[29] Jain S (2022). Insight into the antifungal effect of chitosan-conjugated metal oxide nanoparticles decorated on cellulosic foam filter for water filtration. Int. J. Food Microbiol..

[30] Hosseini F, Sadighian S, Hosseini-Monfared H, Mahmoodi NM (2016). Dye removal and kinetics of adsorption by magnetic chitosan nanoparticles. Desalin. Water Treat..

[31] Saleh MR, Thabet SM, El-Gendy RA, Saleh M, El-Bery HM (2022). MIL-53 (Fe) for constructing hydrogenated Fe_3_O_4_@C@TiO_2_ double core-shell nanocrystals as superior bifunctional photocatalyst. J. Photochem. Photobiol. A Chem..

[32] Eltaweil AS, El-Monaem EMA, Mohy-Eldin MS, Omer AM (2021). Fabrication of attapulgite/magnetic aminated chitosan composite as efficient and reusable adsorbent for Cr (VI) ions. Sci. Rep..

[33] Mokhtari A, Sabzi M, Azimi H (2021). 3D porous bioadsorbents based on chitosan/alginate/cellulose nanofibers as efficient and recyclable adsorbents of anionic dye. Carbohydr. Polym..

[34] Quesada HB (2022). Caffeine removal by chitosan/activated carbon composite beads: Adsorption in tap water and synthetic hospital wastewater. Chem. Eng. Res. Design.

[35] Gong Y (2021). Aminated chitosan/cellulose nanocomposite microspheres designed for efficient removal of low-concentration sulfamethoxazole from water. J. Mol. Liq..

[36] Li J (2017). Preparation and adsorption properties of magnetic chitosan composite adsorbent for Cu^2^^+^ removal. J. Clean. Prod..

[37] Lichtfouse E (2019). Chitosan for direct bioflocculation of wastewater. Environ. Chem. Lett..

[38] Fauzani D, Notodarmojo S, Handajani M, Helmy Q, Kardiansyah T (2021). Cellulose in natural flocculant applications: A review. J. Phys. Conf. Ser..

[39] Rahaman MH, Islam MA, Islam MM, Rahman MA, Alam SMN (2021). Biodegradable composite adsorbent of modified cellulose and chitosan to remove heavy metal ions from aqueous solution. Curr. Res. Green Sustain. Chem..

[40] Zhang D (2019). A three-dimensional macroporous network structured chitosan/cellulose biocomposite sponge for rapid and selective removal of mercury (II) ions from aqueous solution. Chem. Eng. J..

