# Supplementary material for: Bifunctional of Fe3O4@chitosan nanocomposite as a clarifying agent and cationic flocculant on different sugar solutions as a comprehensive semi industrial application
Source: Sci Rep. 2024 Jan 22;14:1848. doi: 10.1038/s41598-024-52111-6 (PMC10803765; doi:10.1038/s41598-024-52111-6)
Supplement: Supplementary file 1 — Supplementary Information. [file 41598_2024_52111_MOESM1_ESM.docx]

**Supplementary materials**

**Bifunctional of Fe_3_O_4_@Chitosan nanocomposite as a clarifying agent and cationic flocculant on different sugar solutions as a comprehensive semi industrial application**

**Hemat M. Dardeer^1^, Ahmed S. Ibrahim^2^, Ahmed N. Gad ^3^, Abdel-Aal M. Gaber^^[[1]](#footnote-1)^*4^**

^1^ Chemistry Department, Faculty of Science, South Valley University, Qena, Egypt

^2^ Faculty of Sugar and Integrated Industries Technology, Assiut University, Egypt

^3^ Research and Development Center of ESIIC, Quos, Egypt

^4^ Chemistry Department, Faculty of Science, Assiut University, Assiut 71516,

Egypt

**Corresponding Author: gaber@aun.edu.eg**


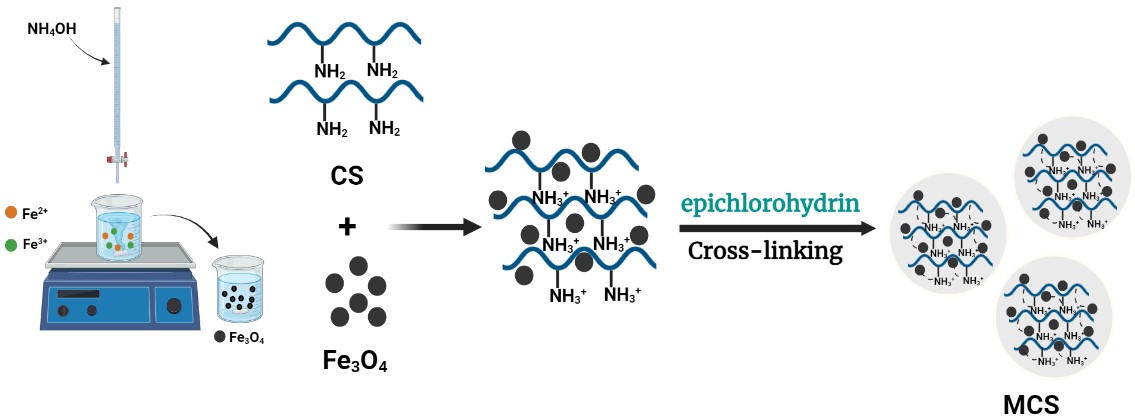


**Figure S1.** Preparation mechanism of MCS nanocomposite.

**
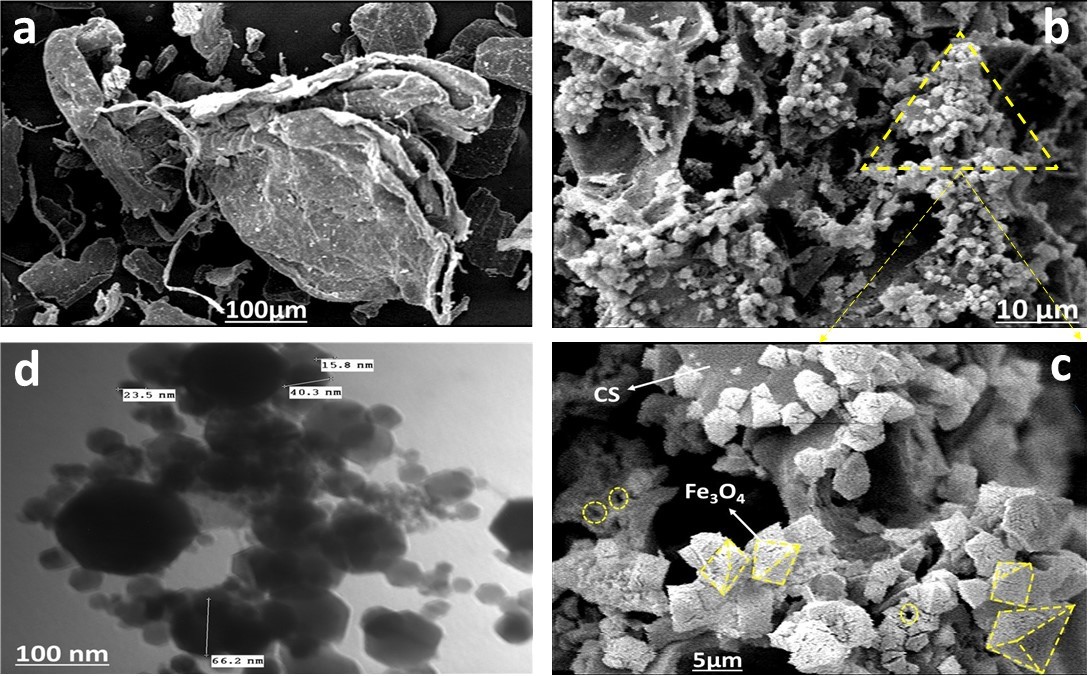
**

**Figure S2.** (a) SEM image for CS, (b) SEM image for MCS, and (c) TEM image for MCS.

**
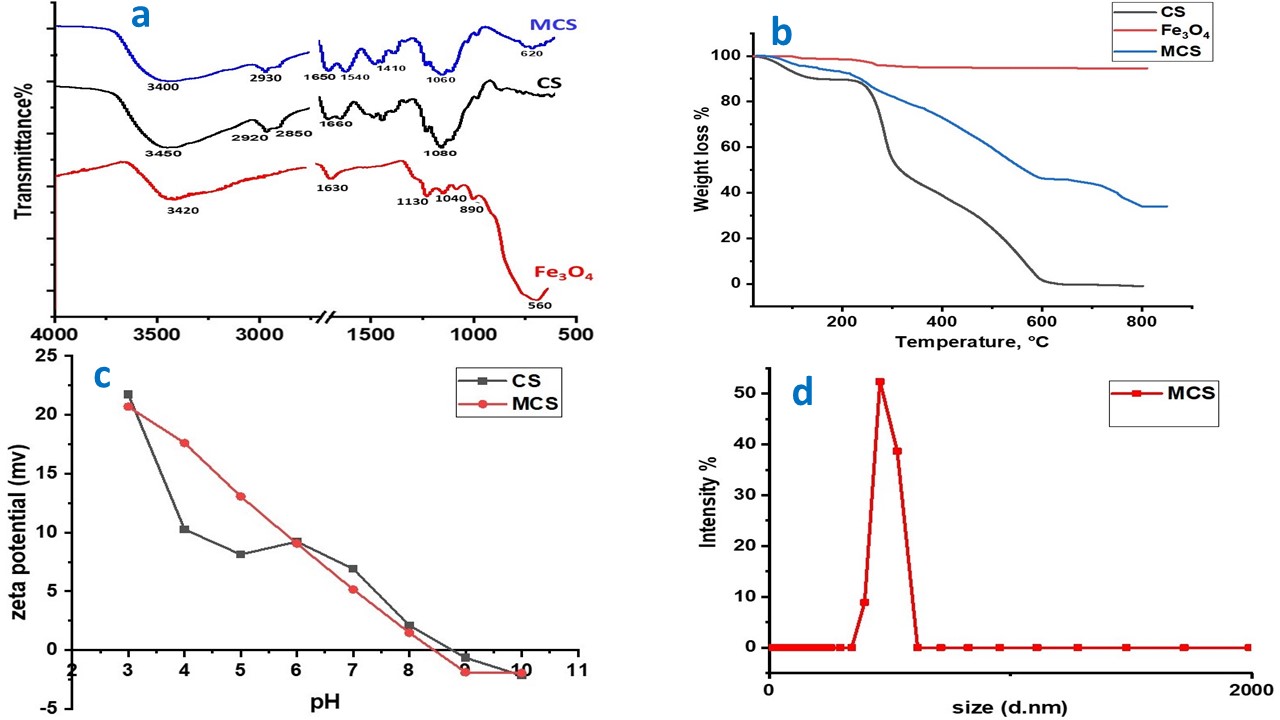
**

**Figure S3.** (a) FT-IR spectra of CS, Fe_3_O_4_, and MCS nanocomposite, (b) Thermogravimetric curves for CS, Fe_3_O_4_, and MCS nanocomposite, (c) Zeta potential curves for CS and MCS nanocomposite, and (d) Particle size distribution of MCS nanocomposite.

**
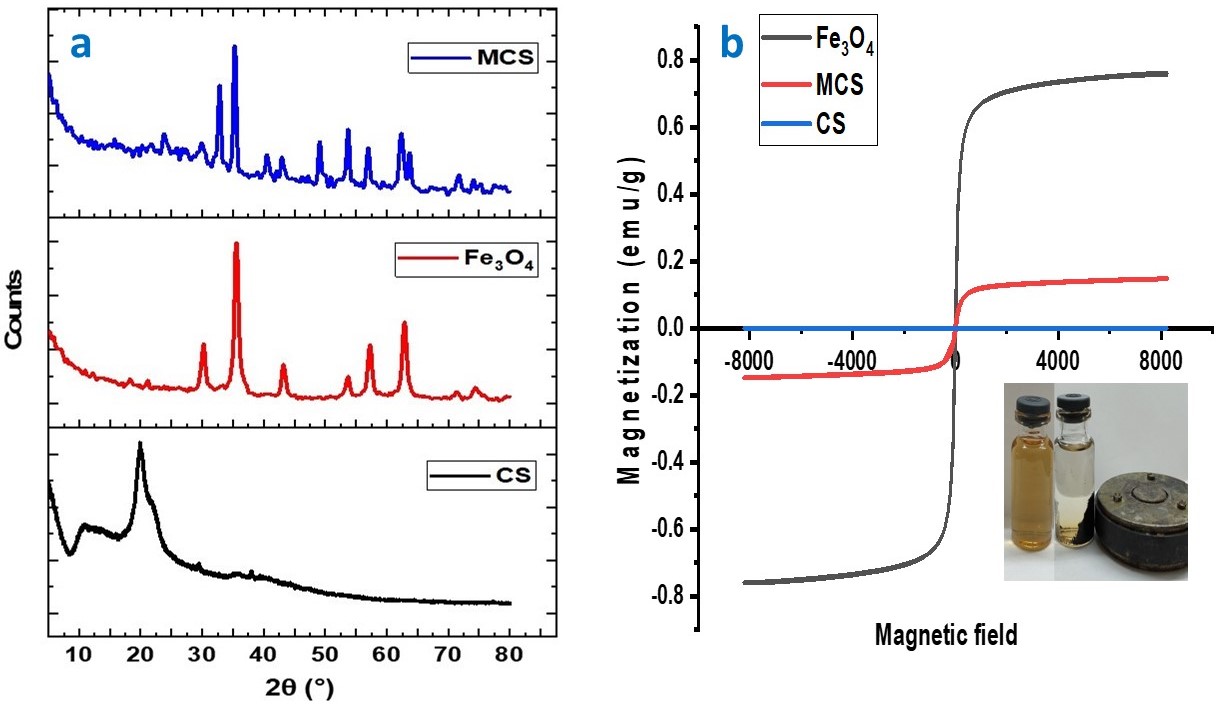
**

**Figure S4.** (a) XRD patterns of CS, Fe_3_O_4_, and MCS nanocomposite, and (b) Magnetization curves for Fe_3_O_4_ and MCS nanocomposite.

**
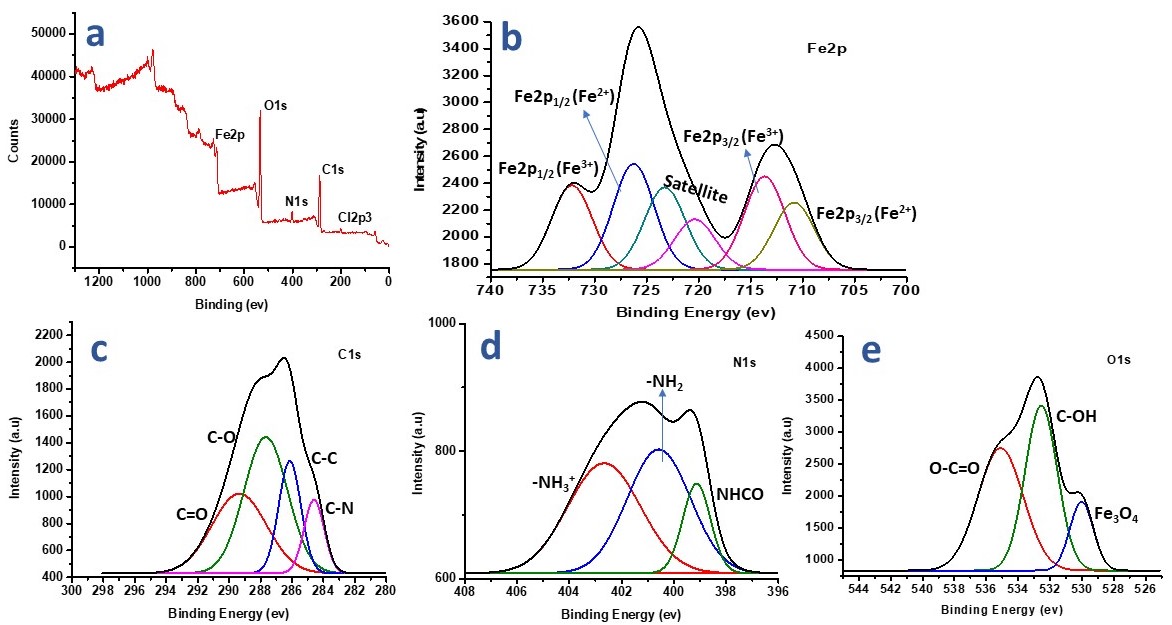
**

**Figure S5.** (a) XPS survey scan for MCS nanocomposite, (b) Fe2p spectrum of MCS nanocomposite, (c) C1s spectrum of MCS nanocomposite, (d) N1s spectrum of MCS nanocomposite, and (e) O1s spectrum of MCS nanocomposite.

**
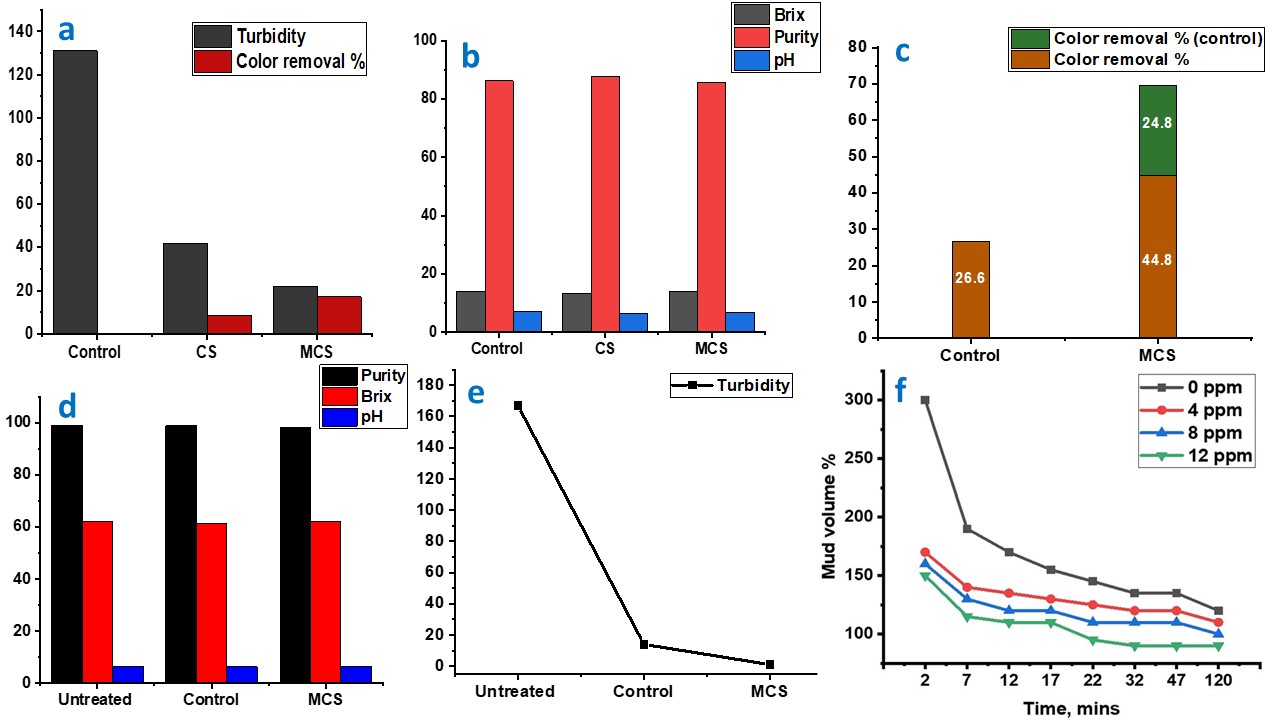
**

**Figure S6. (a)** Comparison between control, CS, and MCS in color removal % and turbidity on CJ, (b) Brix°, purity, and pH performance for control, CS, and MCS on CJ. (c) Comparison between control and MCS in color removal % on sugar syrup. (d) Brix°, purity, and pH performance for untreated syrup, control, and MCS. **(e)** Turbidity curve for untreated syrup, control, and MCS. (f) Flotation rate curves for anionic flocculant dosages against time.

**
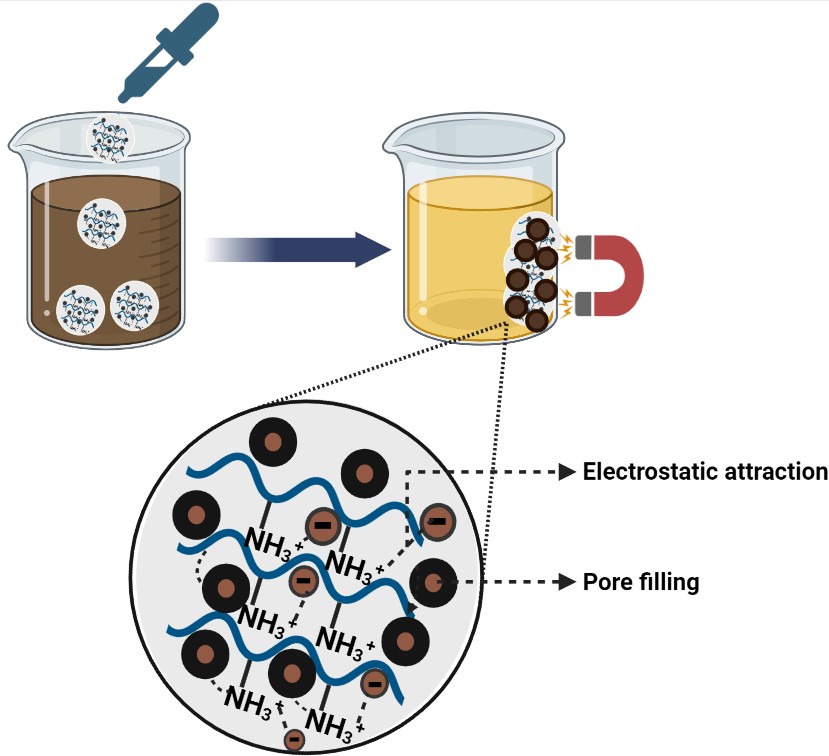
**

**Figure S7.** Adsorption illustration of colorants using MCS nanocomposite and magnetic field.

**Figure S8.** Camera image for (a) untreated syrup, (b) treated syrup with CS, and (c) treated syrup with MCS nanocomposite.

**
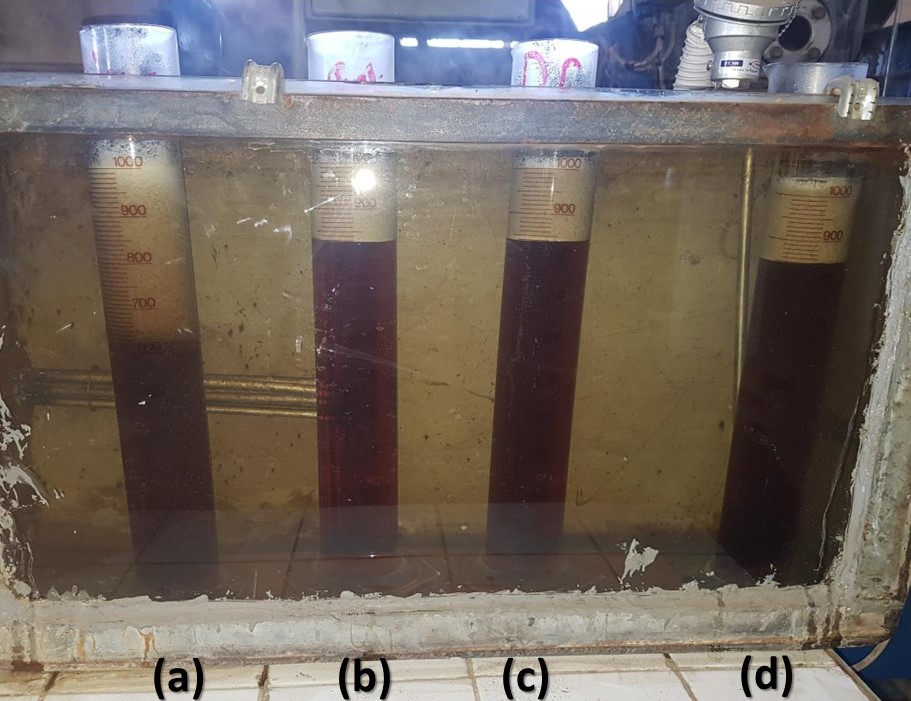
**

**Figure S9.** Mud volume % after 2 mins of adding anionic flocculant dosage, (a) 0 ppm, (b) 4 ppm, (c) 8 ppm, and (d) 12 ppm

**
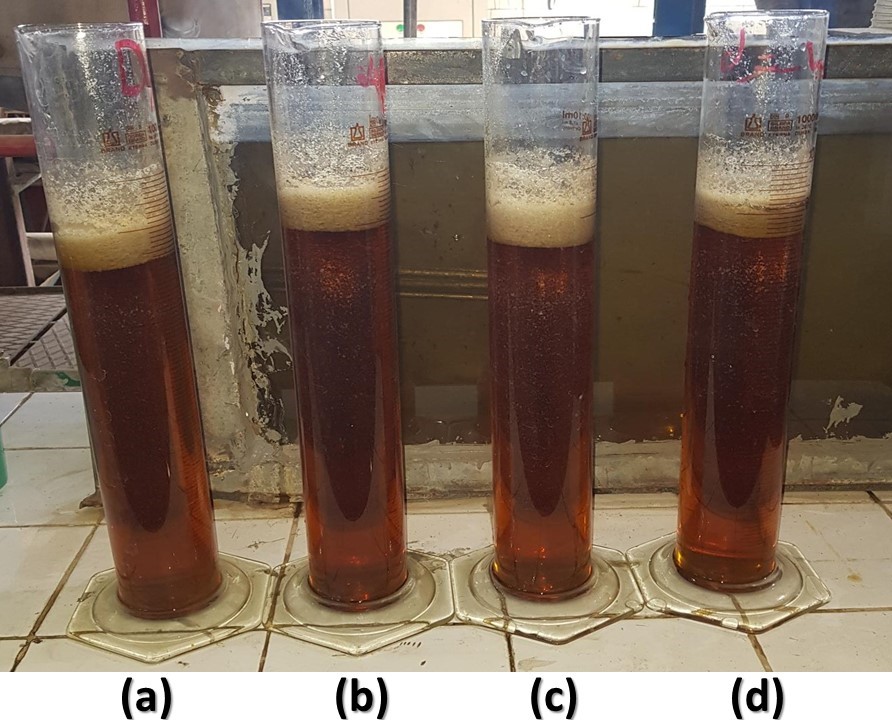
**

**Figure S10.** Mud volume % after 120 mins adding anionic flocculant dosage, (a) 0 ppm, (b) 4 ppm, (c) 8 ppm, and (d) 12 ppm

**Table S1** Specifications of mixed juice (MJ) produced by Quos sugar

Factory

| Parameter | MJ |
| --- | --- |
| Brix | 13.8 |
| Purity | 84.18 |
| P_2_O_5_ | 3.49 |
| Ash % Bx | 3.49 |
| Ca % Bx | 0.41 |
| SiO_2_ % Bx | 1.76 |
| K_2_O % Bx | 1.006 |
| Na_2_O % Bx | 0.056 |
| pH | 5.9 |

**Table S2**

Comparison between control, CS, and MCS on the quality parameters of CJ

| Parameter | Untreated | Control | CS | MCS |
| --- | --- | --- | --- | --- |
| Brix | 13.80 | 13.96 | 13.44 | 14.02 |
| Purity | 84.18 | 86.19 | 87.75 | 85.75 |
| Color% Brix | - | 10422 | 9516 | 8644 |
| Turbidity | - | 131 | 42 | 22 |
| TDS | 2.95 | 3.06 | 3.23 | 3.11 |
| pH | 6.09 | 6.98 | 6.59 | 6.85 |
| Color removal % | - | - | 8.7 | 17.1 |

**Table S3**

Comparison between traditional clarification and MCS on sugar syrup

| Parameter | Untreated | Control | MCS |
| --- | --- | --- | --- |
| Brix | 62.28 | 61.50 | 62.17 |
| Purity | 99.1 | 98.9 | 98.4 |
| Color% Brix | 3324 | 2439 | 1834 |
| pH | 6.6 | 6.4 | 6.6 |
| Color removal % | - | 26.6 | 44.8 |
| Color removal % (control) | - | - | 24.8 |
| Turbidity | 167 | 14 | 1 |

**Table S4**

Mud volume percentages with anionic flocculant dosages

| Time (mins) | Mud volume % with anionic flocculant dosages | | | |
| --- | --- | --- | --- | --- |
|  | **0 ppm** | **4 ppm** | **8 ppm** | **12 ppm** |
| 2 | 300 | 170 | 160 | 150 |
| 7 | 190 | 140 | 130 | 115 |
| 12 | 170 | 135 | 120 | 110 |
| 17 | 155 | 130 | 120 | 110 |
| 22 | 145 | 125 | 110 | 95 |
| 32 | 135 | 120 | 110 | 90 |
| 47 | 135 | 120 | 110 | 90 |
| 120 | 120 | 110 | 100 | 90 |

1. * Corresponding author

   E-Mail address: [gaber@aun.edu.eg](mailto:gaber@aun.edu.eg) (Abdel-Aal Gaber)

   <https://orcid.org/0000-0003-3876-0630> [↑](#footnote-ref-1)
